# Diffusion with Discontinuous Swelling: III. Type II Diffusion as a Particular Solution of the Conventional Diffusion Equation

**DOI:** 10.6028/jres.081A.013

**Published:** 1977-04-01

**Authors:** Anton Peterlin

**Affiliations:** Institute for Materials Research, National Bureau of Standards, Washington, D.C. 20234

**Keywords:** Concentration dependent diffusion coefficient, concentration front, discontinuous swelling, unconventional diffusion, velocity of concentration front propagation

## Abstract

Very often a non-solvent diffuses into a glassy polymer with a steep concentration profile proceeding at an almost constant rate *v* yielding a weight gain proportional to time. Such a diffusion is called type II diffusion in order to distinguish it from the more usual “Fickian” diffusion proceeding without such a constant concentration front and yielding, at least in the beginning, a weight gain proportional to the square root of time. It turns out that the conventional diffusion equation without any special new term but with a diffusion coefficient rapidly increasing with concentration has a series of solutions representing exactly such type II diffusion with *v* as a completely free parameter which determines the steepness of concentration front. With the usual boundary conditions and infinite medium the diffusion coefficient has to become infinite at the highest penetrant concentration. This case can be considered as an extreme limit which is approached to a high degree in an actual experiment. The finite sample thickness, however, requires only a very large but not an infinite diffusion coefficient. Hence type II diffusion is only a special case of possible diffusion processes compatible with the conventional diffusion equation without any need for new terms if only the diffusion coefficient increases sufficiently fast with penetrant concentration.

## 1. Introduction

In recent years a large amount of experimental [1–12] and theoretical [13–21] work was done on diffusion of liquids in polymer glasses with almost discontinuous swelling which is now generally referred to as type II diffusion [2]. It is characterized by three basic conditions: (1) As solvent penetrates the polymer, a sharp advancing boundary separates the inner glassy core from the outer swollen and rubbery shell, (2) Behind the solvent’s advancing front, the swollen gel is in an almost equilibrium swelling state, (3) the boundary between swollen gel and glassy core advances at almost constant rate varying in polystyrene, depending on temperature, penetrant and its activity, between 0.2 and 10 × 10^−6^ cm/s. As a consequence the specific weight gain per unit cross section of the diffusing front, *W* increases almost linearly with time as expected from the almost constant velocity, *v*, of progress of the swelling boundary between the low and high concentration of the penetrant. An effect of minor importance for the diffusion process itself is the partial destruction of polymer with craze and crack formation in the swollen region of the polymer.

As a glassy polymer is cooled the speed of advancing front falls off and a critical point is reached with zero velocity *v.* Type II diffusion is replaced by a more or less conventional Fickian diffusion. In this case the polymer has both a glassy shell and glassy core. The swelling by the penetrant is not sufficient for the reduction of the glass transition point of the swollen material below or to the temperature of the experiment. According to some analyses of the low molecular weight paraffin diffusion into polystyrene, the effect occurs at relatively high penetrant activity [17], between 0.5 and 1, while older data on methyl acetate diffusion into poly(methyl methacrylate) and on acetone diffusion in cellulose nitrate show the linear increase of *W* with time most clearly at very low activity [1a].

Since the advancing of a sharp boundary between low and high solvent concentration at an almost constant velocity is a most unexpected feature of a diffusion process, a new name, type II diffusion was created [2] and the phenomenon tried to be explained by a new term in the diffusion equation depending on the divergence of the stress field originating at the boundary between the conspicuously swollen and the almost nonswollen material [15, 16]. An analysis of the diffusion process in a nonlinear sorption and diffusion range, however, shows that the effect is neither so strange nor so unexpected to need a new name and a new term in the diffusion equation. It follows automatically from the classical formulation of diffusion if the sorption (*S*) and diffusion (*D*) coefficient are strongly dependent on concentration *c* of diffusant or its chemical potential *μ.*

In this paper it will be demonstrated that the general diffusion equation in an infinite medium can have a steady state solution with a time independent concentration profile progressing uniformly through the polymer at a constant velocity. The sole condition for such a solution is that the mobility becomes infinite at the maximum concentration *c*_0_ or maximum chemical potential of the diffusant. This condition is a natural consequence of the geometrical boundaries of the sample extending from −∞ to +∞. A constant current *j* from the boundary at −∞ can be only sustained if *D* increases to infinity while (*dc*/*∂x*)_−∞_ goes to zero. The more realistic consideration of the finite dimensions of the sample removes the need for an infinitely large diffusion coefficient but also makes the mathematics a little more complicated than attempted in this paper.

For the sake of simplicity only the linear case will be treated. The swelling boundary has a constant cross-section and proceeds with constant velocity in the *x*-axis direction. The specific weight gain of the sample per unit time, *dW*/*dt*, is constant as in the case of type II diffusion if one neglects the transients at the beginning and the end of the experiment. The transients are of course completely neglected in the consideration of the steady state boundary propagation through an infinitely thick sample extending from −∞ to +∞.

In the actual membrane the diffusant mobility may increase quite drastically with glass to rubber transformation but still remains finite. Yet the changes are quite substantial, from *D* = 10^−12^ cm^2^/s in a glass to 10^−6^ cm^2^/s in a gel. The above mentioned steady state solution in the infinite medium is therefore the asymptotic approximation to the actual diffusion process if the mobility increases by a few orders of magnitude while the sorbent concentration increases from *c* = 0 to *c*_0_. This is indeed the case with a diffusant which transforms the glassy polymer into a rubbery gel. The gel must be so much lower in polymer content that the sorbate flows through it almost freely and thus easily supplies the amount of liquid requested for the swelling at the propagating concentration front.

Transient from the start at *t* = 0 with *c* = 0 throughout the sample to the steady state solution is a combination with the usual type I diffusion with the initial weight gain proportional to the square root of time. During this transition time the steady state of the concentration tail gets established in front of the almost constant concentration profile. The shape of the profile depends on the propagation velocity [14]. The total weight gain is a sum of two terms, one proportional to *t*^1/2^ and one proportional to *t* [13]. The former soon tapers off while the latter remains practically unchanged. Another transient effect occurs at the end of the experiment when the steadily progressing concentration fronts nearly meet each other in the center of the film if the liquid enters the film from both surfaces. According to Hopfenberg et al. [8, 9], the specific weight gain slightly increases in the case of propagation of *n*- pentane into glassy polystyrene just before the diffusion process is completed. Such an effect finds a simple explanation in the superposition of the concentration tails in front of the concentration discontinuities as they approach the center plane of the film. Hence the concentration at each point between the advancing fronts increases faster than formerly when the fronts were farther apart. As a consequence the critical concentration for the transformation from glass to gel is reached earlier. This shows up in an acceleration of specific weight gain at the end of type II diffusion.

The diffusion equation and the boundary conditions do not impose any limitation on *v* and hence do not determine the velocity of profile propagation. The velocity must be connected with some independent material property. It seems to be a good suggestion that the increase of concentration of the penetrant produces by the ensuing membrane swelling a sufficiently high stress on the polymer network for the rupture or partial disentanglement of most strained chains. But since the chain rupture or the pulling out of chains is not an instantaneous process an effect of this type sufficiently large to permit a substantial swelling is only achieved if the stress persists for a sufficiently long time. This condition determines the propagation velocity of the concentration profile. If the condition cannot be met the transport of permeant proceeds in the usual way as type I diffusion, i.e., without a discontinuous concentration front and with a weight increase which is for a long while proportional to the square root of time.

## 2. Mathematical Description of Type II Diffusion

All the theoretical work up till now is purely descriptive without any serious attempt of explanation. Peterlin [13, 14] based his description on the diffusion and sorbate concentration dependence on time in front of a sorption discontinuity, i.e., a jump from c* to *c*_0_, moving through the sample at constant velocity *v.* In this area the Fickian diffusion equation reads [13]

$$\frac{\partial }{\partial x^{\prime }}\left(D\frac{\partial c}{\partial x^{\prime }}+vc\right)=\frac{\partial c}{\partial t}$$ (1)

with the diffusion current

$$j^{\prime }=-D\frac{\partial c}{\partial x^{\prime }}-vc.$$ (2)

Here *x′ = x − vt, D* is the constant diffusion coefficient, and c* the maximum concentration of liquid in the glass beyond which a discontinuous transformation to a gel with concentration *c*_0_ and so high diffusivity takes place that practically no driving gradient is needed for the supply of liquid to the proceeding discontinuity front. The point *x* corresponding to a constant *x*′ moves with the constant velocity *v* to larger *x*′ as does the concentration discontinuity. The Fickian formulation with the current proportional to the negative gradient of concentration instead of that of chemical potential is fully legitimate because the sorption coefficient is assumed constant although much smaller in front than behind the concentration discontinuity.

Behind the discontinuity the sorbate concentration is practically constant (*c*_0_) and hence the current *j*′ in the *x′,t* frame equal to zero. In the laboratory system *x,t* the diffusion current *j* is by *vc* larger than *j′.* Hence it turns out to be *−D∂c/∂x* in front of discontinuity and *vc*_0_ behind it. Exactly speaking the very large value of *D* in the swollen region permits even a higher value of current than *vc*_0_ in order to supply the liquid needed for the gradual establishment of the steady state concentration tail in front of discontinuity.

From these equations one obtains the weight gain per unit cross section of the film as function of time

$$\begin{array}{cc} W=c*{\left(2D/\pi \right)}^{1/2}t^{1/2}+c_{0}vt & \;v^{2}t/4D\to 0 \end{array}$$ (3)

for small *t* and

$$\begin{array}{cc} W=c*D/v+c_{0}vt & \;v^{2}t/4D\to \infty \end{array}$$ (4)

for large *t* after the concentration profile in front of the discontinuity has reached its stationary value. The concentration is measured in *g* of sorbate per cm^3^ of sorbent.

The first term describes the effect of Fickian diffusion with constant diffusion coefficient *D* in front of the discontinuity which after the initial proportionality to *t*^1/2^ reaches the constant value c*D/v. The second term describes the weight gain in the highly swollen region behind the discontinuity proportional to the uniform increase of the volume of the swollen region as a consequence of constant velocity *v* of the profile propagation. In these expressions one assumed that the diffusivity in the gel is so much higher than in the glass that there practically no measurable gradient is needed for the transport of the liquid. A rather similar approach was used by Crank [22] in his description of different cases of diffusion in solids.

**Figure 1:**
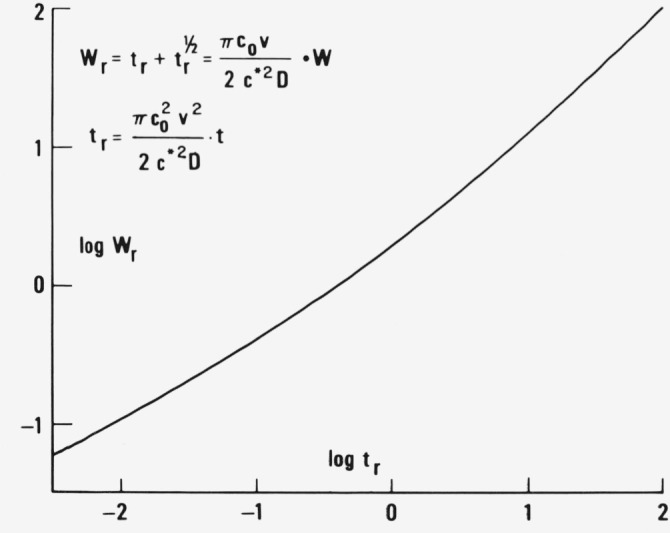
Bilogarithmic plot of weight gain *W* over time *t* according to eq (3).

In the initial state the coexistence of the square root and linear term in time according to eq (3) yields over appreciable time intervals a constant power *m* relationship between weight gain and time, *W* = *Bt^m^*, as can be seen from figure 1. where log *W* is plotted versus log *t.* At small *t* one has the square root, *m* = 0.5, and at high *t* the linear, *m* = 1, dependence. A rather constant *m*, i.e., a rather constant slope, applies to intervals extending over almost two decades of time. One can guess that within experimental errors such a theoretical prediction may sufficiently well fit the power law of weight gain observed in some cases of unconventional sorption [7].

There is no limitation about *v* in eqs 1 to 4 although the time dependence of the concentrations profile in front of the concentration discontinuity [14] and the asymptotic steady state value c*D/*v* depend on *v.* The dimensionless time parameter *a* = (*v*^2^*t*/*4D*)^1/2^ increases and the total equilibrium sorption c*D/*v* in that region decreases with increasing *v*. The constant rate progression of the concentration discontinuity yielding the linear weight gain according to eqs (3) and (4) characteristic for type II diffusion hence is a distinct possibility compatible with the classical diffusion equation as long as the supply of the sorbate through the highly swollen section of the film is sufficiently high. This flux has to fill continuously with the solvent a volume increasing in depth by *v* per second in spite of the steadily increasing length of the supply route from the outer surface of the film to the steadily progressing discontinuity. In all practical cases that requires that the diffusion coefficient of the gel (*D*_2_) is some orders of magnitude higher than in the not swollen glass (*D*_1_). In the ideal case of an infinite extension of the film in the direction perpendicular to the front this condition amounts to an infinite value of *D*_2_.

One concludes that the type II diffusion can be sufficiently well approximated on the basis of the classical diffusion equation by the limiting case of constant rate propagation of a concentration discontinuity. The very beginning (*t* = 4*Da*^2^/*v*^2^ with *a* → 0) with the weight gain proportional to *t*^1/2^ may be over in such a short time that it is practically unobservable. After that a linear increase of weight gain with time is established. In a sample with a very large distance between the border where the liquid enters and the concentration discontinuity the weight gain must show some incipient drop as soon as the influence of diffusion time through the swollen region becomes preceptible on the solvent supply at the concentration discontinuity. This does not occur in the above-quoted experiments by Hopfenberg et al. [7–9], where for a polystyrene (PS) film of 38*μ*m thickness the diffusion from both surfaces of the film was completed between 12 min and 400 hours, dependent on temperature, activity of *n*-pentane vapor, and on polymer orientation. The velocity of concentration front propagation in the same cases varied between 1.3 × 10^−9^ (cast annealed PS, 25 °C, *n*-pentane gas activity 0.63) and 2.65 × 10^−6^ cm.s^−1^ (biaxially oriented PS, 35 °C, activity 1). Since the maximum liquid concentration was about 13 g per 100 g of polymer one needs indeed such an extremely small gradient for steady supply of so small an amount of liquid increase per unit time that its decrease would be hard to detect.

Hence the contrast between types I and II diffusion is one of the consequences of the extreme nonideality of transport properties in the latter case. A discontinuous substantial increase of sorption and diffusion takes place after a limiting concentration c* of sorbent is attained in the film by more or less conventional type I diffusion. The constant rate propagation of the concentration discontinuity represents a pseudostationary state of diffusion in such a nonideal medium. The velocity *v*, however, is not derivable from the diffusion equation and boundary conditions and must be determined by some other mechanism of solvent-polymer interaction.

The non-ideality of the medium, i.e., an almost discontinuous increase of diffusion coefficient as soon as the penetrant concentration reaches a critical value c* was already suggested by King [1] for explanation of the unconventional diffusion of alcohol vapor into wool and keratine. His results cannot be directly compared with eqs (1 to 4) because he uses an expansion of *D* in powers of *c* which makes the results more complicated, dependent on the coefficients of the power expansion. But he actually estimates the effect of rapidly increasing *D* with concentration yielding a steep concentration front propagating with almost constant velocity into the medium.

Frisch et al. [15, 16] assume that the diffusion current density is caused by the gradient of chemical potential and the divergence of the partial stress tensor of the penetrant

$$j=-Bc[\partial \mu /\partial x-(1/c)\partial S_{xx}/\partial_{x}]$$ (5)

if one puts

$$S_{xx}=s\int_{0}^{x}c(x^{\prime },t)dx^{\prime }$$ (6)

i.e., if one makes the assumption that the partial stress tensor *S* of the penetrant at any location and time is proportional to the total uptake of the penetrant up to that location and time. With a constant proportionality factor s one obtains from

$$\partial S_{xx}/\partial x=sc(x,t)$$ (7)

a current

$$\begin{array}{l} j=-Bc\partial \mu /\partial x+Bsc=-Bc(x,t)(\partial \mu /\partial x-s)\\ \;=-Bc\partial \mu /\partial x+vc. \end{array}$$ (8)

With constant *D* and *μ* = *RT ℓ*n *c* one obtains

$$c(x,t)=(c_{0}/2)\{\exp(xv/D)\text{erfc}[(x+vt)/2{\left(Dt\right)}^{1/2}]+\text{erfc}[(x-vt)/2{\left(Dt\right)}^{1/2}]\}$$ (8a)

for diffusion into the half space with *x* ⩾ 0. According to this solution the steepness of concentration “discontinuity” at *c*_0_/2 soon tapers off in exactly the same manner as in conventional diffusion. The point with *c = c*_0_/2 proceeds into the medium at almost constant velocity *v.* Points with smaller *c* move faster and those with higher *c* move more slowly so that the maximum slope, located at *c = c*_0_/2, gradually diminishes and finally becomes zero. The weight gain *W*/*c*_0_ is initially proportional to *t*^1/2^. With increasing time it approaches strict proportionality to *t.* The assymptote goes exactly through the origin of *W,t* coordinate system.

The “theory” depends on the “natural” assumption formulated in eq (6) that the partial stress tensor of the liquid *S_xx_* in the highly swollen section of the sample linearly increases with *x* and hence goes to infinity in an infinite medium. Since to our knowledge no such stress exists one gets the impression that all the naturalness of the above assumption is rather pragmatic based on the success, i.e., on the yielding of a constant term in eq (5) one wants to have in order to fit the experimental data. There is no correlation between any property of the polymer or penetrant and the coefficient *s* which together with *B* determines the velocity of propagation *v* = *Bs* of the concentration front. Hence the derivation of the authors, the introduction of the new term depending on the stress gradient, and the choice of stress which yields the constant component *Bsc* of the current can be labelled mathematical description but not explanation of the type II diffusion.

The difference between the solution of Frisch et al. and that of Peterlin is in the shape of concentration profile and in limiting weight gain. According to the former concept the shape of concentration front gets gradually less steep and finally becomes perfectly flat as at the end of conventional diffusion. In the latter formulation it rather soon reaches a finite shape which after that does not change any more. The asymptotic weight gain is strictly proportional to time in the former case and still contains a square root term in time in the latter case.

None of the two concepts yields a front propagation containing linear and square root terms in time. Both yield a constant or almost constant velocity of propagation of concentration front if this is taken as the point of fastest concentration increase, i.e., at *c = c*_0_/2 (model of Frisch), or at the point where the concentration jumps from c* to *c*_0_ (model of Peterlin). Hence the experimental data of Kwei and Zupko [6] yielding such a combination of linear and square root of time terms cannot be described by any of the two concepts.

A further difficulty for the model of Frisch et al. shows up in desorption experiments [4, 8] which correspond to purely Fickian diffusion. Peterlin’s formulation is not affected because it only describes the diffusion process if a concentration front moves with a constant velocity through the medium. If there is none the diffusion is conventional. Frisch’s formulation, however, starts with a large *S_xx_* which at completed sorption increases linearly from 0 at the first boundary, *x* = 0, to *sc*_0_*d* at the other boundary, *x = d.* The gradient *∂S_xx_*/*∂x* would simply continue to pump the liquid through the film at the same rate *v* and in the same direction as during the preceding type II diffusion. There is no end to the process. The way out of this impass is the simultaneous consideration of the identical type II diffusion pumping the liquid at the same rate *v* and in opposite direction from the other boundary as a consequence of *S_xx_*(*d*) starting with value 0 at *x = d* and increasing to *sc*_0_*d* at *x* = 0. At the end of sorption the sum of *S_xx_*(0) and *S_xx_*(*d*) is constant, *sc*_0_*d*, throughout the sample. Its gradient disappears and so does the current corresponding to type II diffusion. This happy end effect has however the disadvantage that it yields the same negative result for all previous and later times. The sum of both *S_xx_* is a constant as far as *x* is concerned although it increases with time during sorption and decreases during desorption as does the total uptake of penetrant. Hence it cannot generate any steady flow as observed in type II diffusion. The modification of *S_xx_* in eqs (5) and (6) which explains the end of sorption and the Fickian type of desorption process excludes the explanation of the unconventional sorption the equations were formulated for.

The formulation according to eq (5) leads to another quite unexpected consequence. The coefficient s in the stress tensor, eq (6), which multiplied by *B* yields the velocity of concentration front propagation seems to be a constant of the penetrant-polymer system and hence independent of *c.* All the concentration dependence is in the factor *B* which affects equally the type I (the first term) and type II (the second term) diffusion. Hence *v* is proportional to the conventional diffusion coefficient. The proportionality factor *s* depends on the penetrant-polymer combination but not on concentration. That leads to the peculiar consequence that the normal type of diffusion is not the classical “Fickian” diffusion but the type II diffusion.

In contrast to that the experiments by Hopfenberg et al. [7, 8] on the dependence of type II diffusion on vapor activity convincingly show a rapid decrease of *v* with decreasing activity a and the complete cessation of such diffusion below a limiting activity a* ~0.3. Hence s cannot be a constant but must be a function of *a*-a* vanishing at a* and for any a below a*. Although this dependence is the crucial point of explanation of type II diffusion it was never attempted to be derived from material properties.

Moreover the presence of the term *B_s_* in diffusion equation completely changes the concentration increase in time ahead of the concentration front as soon as the local concentration c surpasses the value c* corresponding to the limiting activity a*. This consequence may be less disturbing if one assumes that ahead of concentration front c is always smaller than c* and behind it larger than or equal to c*. But it still may cause some problems in the front itself if the concentration does not jump discontinuously from *c*_1_ = c* ahead of the front to *c*_2_ behind the front. The variation of propagation velocity with *c* must create a special type of front profile which was never yet analyzed from this point of view. Without any more detailed analysis one can only guess that any concentration increase in the front will become steeper until it will be almost discontinuous.

## 3. Constant Rate Propagation of Concentration Profile

On the basis of the general diffusion equation with *S* and *D* dependent on chemical potential one can easily formulate the conditions for a constant rate propagation of a concentration profile

$$c(x,t)=C(x-vt)=C(x^{\prime }).$$ (9)

The coordinate system *x*′ moves to the right with the same velocity *v* as the concentration profile. Hence in this system the concentration *C* is constant at each *x*′ and the current density *j′* = 0. With these assumptions the solution of the diffusion problem is truly conventional without a need for any additional more or less arbitrary term in the diffusion equation.

According to the thermodynamics of irreversible processes the diffusion current density in the laboratory fixed system *x* is proportional to the chemical potential gradient of the diffusant

j=−(c/f)∂μ/∂x=−DS∂p/∂x=−P∂p/∂x (10)

where *f* is the frictional resistance yielding the diffusion coefficient *D* = *RT*/*f.* The chemical potential *μ* can be expressed as function of vapor pressure *p*(*x,t*) in equilibrium with the sorbate at position *x* at time *t*

$$\mu =RT\;\ell n\;p(x,t)+\mu^{0}$$ (11)

The constant *μ*^0^ is still a function of *T* and of any other parameter independent of *p* and *x.* The concentration *c* is a product of sorption *S*(*p*) and pressure. The conservation of mass yields the concentration variation with time

∂c/∂t=∂(Sp)/∂t=−∂j/∂x=∂(P∂p/∂x)/∂x. (12)

In this well-known classical one dimensional diffusion equation the sorption *S*, the diffusion coefficient *D* and permeability *P* are functions of *p* or concentration *c* and not constants as assumed in the ideal Fickian case. But they are independent of time and location. The membrane is homogenous and does not change although at each point *x* its swelling and diffusivity vary drastically with time. There is also no temperature effect considered.

The drastic change of sorption in the transition from glass to gel implies quite a substantial swelling of the polymer causing eventually the formation of macroscopic cracks as observed in the earlier experiments [2, 3]. For simplicity sake, these dimensional changes are not at all considered in the above formulation of the diffusion equation. Such an omission affects the numerical results hut not the functional properties of the solutions as for instance the existence of the steady state solution and the conditions for a constant velocity of profile propagation.

The thermodynamic equilibrium correlation between concentration and vapor pressure permits to express the concentration profile according to eq (9) as a profile of vapor pressure proceeding with a constant velocity *v*

p(x,t)=q(x−vt) (13)

because the material property *S*(*p*) is independent of *x* and *l*. Hence the value *p*(*x,t*) can be expressed as functions of a single variable *x*′ = *x − vt.* That means that the partial derivatives in eq (10) can be expressed as total derivatives by *x*′. In such a case the differentiation on time is equivalent to differentiation on location

$$\partial q/\partial t=-v\;dq/dx^{\prime }$$ (14)

which with constant *v* transforms eq (10) into the straightforwardly integrable total differential equation

$$\frac{d}{dx^{\prime }}\left(-P\frac{dq}{dx^{\prime }}-vSq\right)=\frac{dj^{\prime }}{dx^{\prime }}=0.$$ (15)

This formulation actually means that there is neither a current nor a change of concentration since the coordinate system *x*′ travels with the same velocity *v* as the concentration or vapor pressure profile. This yields

$$vdx^{\prime }=-D(q)\;dq/q$$ (16)

with the solution

$$-vx^{\prime }=\int_{q(0)}^{q(x^{\prime })}D(q)d\ln\;q.$$ (17)

The boundary condition *q* = *p*(+ ∞) = 0 at *x*′ = + ∞ is met automatically with a finite, not vanishing *D* at *q* = 0. The finite *q* = *p*_0_ at *x*′ = − ∞ demands an infinite value of *D*(*p*_0_). Equation (17) is completely general not imposing any condition on the choice and continuity of *D* beyond those mentioned in connection with the boundary condition, i.e., at *x*′ = − ∞. Note that the value of *S* and its dependence on *p* or *c* do not enter eqs (16) and (17) explicitely.

**Figure 2:**
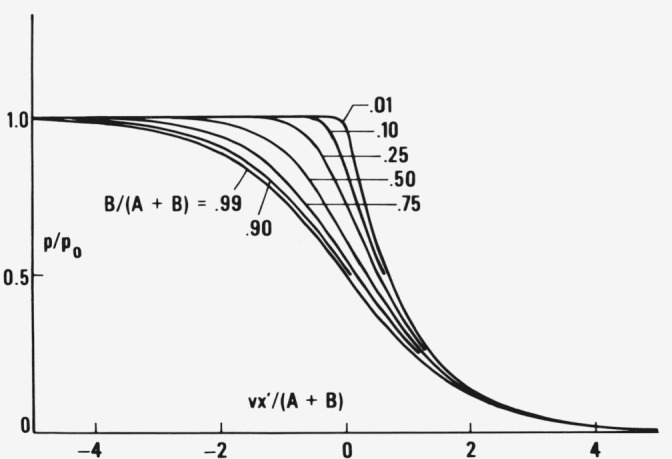
Pressure profiles according to eq (19) moving to the right with constant velocity *v* for different values of *B*/(*A* + *B*) as functions of *vx*′/(*A* + *B*). The smaller the increase of diffusivity with *p* as measured by *B*/(*A* + *B*) the steeper is the profile in this representation.

**Figure 3:**
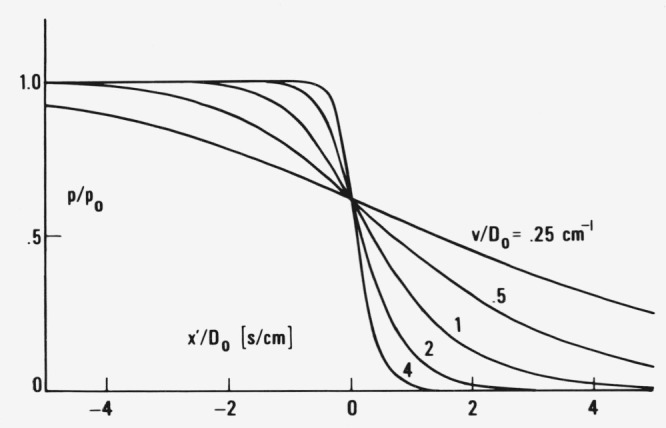
Pressure profiles according to eq (19) moving to the right with constant velocity *v* (parameter of the curves) for *B*/(*A* + *B*) = 0.5 as functions of *x*′/(*A* + *B*) 1 = *x*′/*D*_0_.

One sees that in an infinite medium the diffusion eq (10) permits solutions with a constant concentration or thermodynamically equivalent pressure profile moving with a constant velocity *v* if only the diffusion coefficient goes to infinity at the maximum pressure *p*_0_ applied at the infinitely distant negative boundary *x*′ = − ∞ of the medium. The profile *q*(*x′*) depends on *D*(*p*) and *v* as shown in eq (17). Fora very simple dependence of *D* on *p*

$$D=A+Bp_{0}/(p_{0}-p)$$ (18)

yielding *D*_0_ *= A* + *B* at *p* = 0 and *D* = ∞ at *p = p*_0_, i.e., at the maximum pressure of the sorbate at the infinitely distant film boundary, *x*′ = − ∞, one obtains the profile

$$\ell n\;(q(x^{\prime })/p_{0})-(B/D_{0})\;\ell n\;(1-q(x^{\prime })/p_{0})=-vx^{\prime }/D_{0}+\text{const}.$$ (19)

shown in figure 2 as function of *vx′*/*D*_0_ *= y* for different values of *B*/*D*_0_. If the constant is equaled to zero one only displaces the *q*(*x′*)/*p*_0_ curves horizontally. This does not affect their shape which is our primary interest. The larger *v* and the smaller *D*_0_ the steeper the true profile if plotted against *x*′ (figure 3). The constant pressure profile *q* starts at high positive *y* with a long tail which only slightly differs from that calculated formerly [14] for a constant *D*_1_ continues with a sharp rise of pressure and asymptotically approaches the limiting pressure *p*_0_ at *y* = − ∞. The time independent concentration profile is obtained by multiplication of *q* by *S*(*p*) which exhibits a large increase with *p* approaching *p*_0_ if one wishes to describe the transition from a glass at *p* = 0 (*S* small) to a highly swollen gel with *c = c*_0_ at *p = p*_0_ (*S* large). Hence the concentration profile is expected to vary substantially faster than the pressure profile.

**Figure 4:**
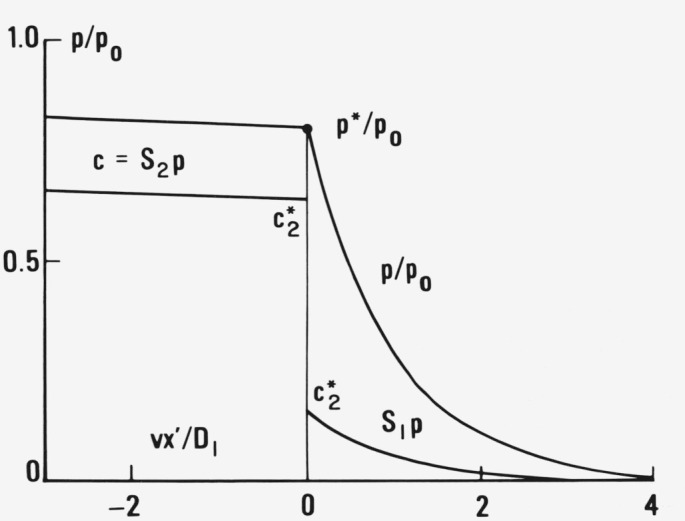
Pressure and concentration profile moving to the right with constant velocity *v* as functions of *vx*′/*D* for a discontinuous change of diffusion constant from *D*_1_ to *D*_2_ = 100 *D*_1_ and of sorption from *S*_1_ to *S*_2_ = 4*S*_1_ at *p* = p* = 0.8 *p*_0_. Note the continuity of *p* and the discontinuity of *c* at the boundary between glass and gel. The constancy of *D* and *S* in the glass (to the right) and in the gel (to the left) yields *p = p*_0_ at a finite value *x′*_0_ *= D/v* instead of at x′_0_ = − ∞ as postulated by the steady state solution (eqs (9) and (13)). As a consequence the propagation velocity *v* has to slow down as soon as *x′*_0_ − *vt* approaches and reaches the outer boundary of the medium by which the penetrant enters because from that moment on the diffusion through the gel is unable to supply the necessary amount of diffusant. Note that one has assumed that up to this time the equilibrium pressure at the entrance to the medium rises continuously and at a constant rate from p* to *p*_0_.

The shape of the profile can be varied as freely as the dependence of *D* and *S* on *p.* In particular, one can choose constant *D* and *S* in the glass and rubber with a discontinuity at p* thus producing a change of slope of *P* and a step-like concentration profile at p* (figure 4) as already partially treated by Crank [22]. Hence the case treated formerly [13, 14] with two concentration independent diffusion constants, *D*_1_ ≪ *D*_2_, is contained in the present more general treatment.

The boundary between unswollen and swollen region moves with the constant velocity *v.* The value of *v* is still completely free and is not at all determined by the diffusion equation or the boundary conditions. From the uniform translation of the concentration profile one derives the weight gain

$$w=j(-\infty )\cdot t=vSp_{0}t=vc_{0}t$$ (20)

proportional to time in perfect agreement with the observations of the steady state of type II diffusion.

One hence has the rather unexpected result that the same mathematical formalism (eq (12)) yields both types of diffusion: Type I for constant or approximately constant and Type II for extremely pressure or concentration dependent *D*, 5, and *P.* Actually the diffusion constant must be infinite for *p = p*_0_ in order to satisfy the boundary condition at *x*′ = − ∞. From a pure mathematical point of view the values of *S* are irrelevant and can be chosen at will. Type I diffusion is a very special case confined to ideally linear systems with constant *D* and *S.* Type II diffusion is more or less close to the diffusion in actual polymer-sorbent systems with *D* and *S* rapidly increasing with *p*, after the initial transient has abated and before the great transport length from the boundary to the moving front starts to reduce perceptibly the velocity of front propagation.

The diffusion coefficient *D* of the penetrant or permeability *P* of an actual medium certainly may become very large but can never assume infinite values. In going from a glass to a swollen rubber or gel the increase in *D* may be many orders of magnitude, from 10^−12^ to 10^−6^ cm^2^/s, so that the steady state solution with *D*(*p*_0_)/*D*(o) ~ ∞ is a very good asymptotic approximation of the actual material transport. But the finite maximum value of *D* imposes a reduction of the permeant supply with increasing transport length *l_tr_* because the pressure gradient at the outer boundary of the sample, (*dp*/*dx*)*_x_*_=0_ ~ (*p*_0_ − *p*(*ℓ_tr_*))/*ℓ_t_*, decreases with this length. That means a slow but steady decrease of propagation velocity of the concentration profile with increasing distance of the profile from the outer boundary of the sample. In a very thick sample the initially constant velocity propagation of the profile and hence the linear increase of weight gain with time are expected to show an observable decrease. But since the most precise measurements were made on extremely thin films with total liquid path less than 0.1 mm one was never faced with this limitation.

A very instructive general picture of the effects caused by the finite although very large diffusion coefficient *D*_2_ in the highly swollen material can be derived from the very schematic figure 4. It is based on a constant *D*_2_, independent of concentration which varies from c*_2_ at the concentration front to the maximum *c*_0_ in thermodynamic equilibrium with the penetrant pressure *p*_0_ at the outer boundary of the sample. In order to simplify the matter one assumes in that which follows that the profile shown in figure 4 is established immediately, at time *t* = 0, at the outer boundary, *x* = 0, so that the initial transient effects can be completely neglected. At *t* = 0 one hence has *x*′ = *x − vt* = 0.

One first notices that the concentration *c*(0) at the outer boundary has the initial value c*_2_ which steadily increases with time up to the maximum value *c*_0_. If one assumes that during this time the concentration front moves with a constant velocity *v* one deduces a slight increase of current density and a more than linear increase of weight gain with time

$$\begin{array}{l} \;j=vc{*}_{2}/(1-v^{2}t/D_{2})\\ \;W=-(c{*}_{2}D_{2}/v)\;\ell n\;(1-v^{2}t/D_{2})\\ \;=c{*}_{2}vt\;(1+v^{2}t/2D_{2}+\cdots )\\ c(0)=c{*}_{2}/(1-v^{2}t/D_{2}). \end{array}$$ (21)

The parameter *v*^2^*t*/*D*_2_ remains small during the whole experiment. Its maximum value is reached at the moment when the front hits the opposite boundary of the sample or meets the front proceeding from this boundary. In the latter case one derives from film thickness *d* and *t*_max_ *= d*/2*v* the maximum value of this parameter

$${\left(v^{2}t/D_{2}\right)}_{\max}=d^{2}/4D_{2}t_{\max}.$$ (22)

In the diffusion of *n*-pentane into cast annealed PS film [8] the film thickness was 38 *μ*m = 3.8 × 10^−3^ cm, *t*_max_ = 60 hours = 2.16 × 10^5^s for *p*_0_ = 550 mmHg at 30 °C. From these data one obtains maximum values for the parameter between 1.7 × 10^−4^ and 1.7 × 10^−2^ if *D*_2_ varies between 10^−7^ and 10^−9^ cm^2^s^−1^. In biaxially oriented film at penetrant activity 1 and *T* = 35 °C the values are up to 300 times higher so that a sufficiently small value of the parameter is only obtainable with the higher diffusion coefficient which is indeed more probable than the lower limit. One can be rather certain that in most cases the parameter is so small that the current *j* is practically constant, the weight increase *W* almost linear with time, and a very small difference *c*(0) − c*_2_ required for a constant supply of liquid to the progressing concentration front. The small value of the parameter also tells that the ideal case, eqs (9) through (20), is a good approximation of the actual polymer-penetrant systems displaying type II diffusion.

But the finite increase in time of penetrant concentration at the sample boundary in contact with the liquid or gas seems to be an important feature of type II diffusion. It is a consequence of the fact that the polymer glass simply cannot expand instantaneously to such an extent that the equilibrium concentration of penetrant could be accommodated. Since the polymer is quite inhomogeneous on molecular scale it exhibits a wide variation of penetrability in very small regions. The more penetrable areas expand substantially more and faster than the less penetrable sections. The forces exerted by the penetrant on taut molecules connecting the latter sections across the former regions will either rupture these chains or pull them out slowly. Both effects require a finite time. As a consequence of such enforced polymer expansion the sample crazes and even cracks. One gets convinced that just this relaxation of the specimen under the osmotic pressure of the penetrant determines the velocity of propagation of the concentration front which is independent of *D* and of any other parameter of the diffusion equation.

In a more realistic approach the inability of the polymer to expand sufficiently for the accommodation of the equilibrium amount of penetrant most likely starts at a substantially smaller concentration than c*_2_, let us say c*_1_. This means that at low activity with *c*(0) < c*_1_ the sorption and diffusion are conventional without any concentration front propagating at constant velocity through the glass. If, however, the activity is so high that *c*(0) > c*_1_ a finite time is needed for proper polymer expansion thus creating the circumstances observed with type II diffusion. The higher the driving force *c*(0) − c*_1_ as compared with c*_1_ and the higher the volume change with the linear expansion of the polymer the more rapidly the glass adjusts to the space requirements of the penetrant, i.e., the higher the velocity of concentration front propagation in perfect agreement with observations.

On the other hand, the steady state profile cannot be established instantaneously. Even if a constant propagation rate is imposed it takes a certain time before the pressure or concentration distribution in the frontal tail assumes time independent values. This was explicitly demonstrated for a concentration discontinuity moving with a constant velocity [14]. The result can be generalized for any profile. In first approximation the weight gain in this transient is proportional to the square root of time. The duration of a substantial contribution of the transient can be unobservably short so that the weight gain does not exhibit a significant initial component proportional to the square root of time but instead is directly proportional to time.

## 4. Conclusions

The steady state solution of the simple diffusion equation with the constant rate of propagation of the fixed concentration profile according to eq (9) is a good approximation of the pseudo-stationary situation of Type II diffusion. Its range is between the usual initial transient with concentration and weight gain dependence on *t*^1/2^ and the final stage with the weight gain less than proportional to *t* because at fixed maximum values of *D* and *P* the sorbate has to be transported to the concentration front through a steadily increasing depth of the fully swollen film. The large ratio between *D*_max_ and *D*_min_ and the small maximum linear extension *ℓ_tr_*, max of films through which the material transport takes place may make very short the duration and rather difficult the observation of the initial and final stages. Therefore, as a rule, the scene is dominated by the pseudo-stationary type II diffusion as formulated in eqs (9) and (19). In the case of thin film with the liquid entering from both plane surfaces the superposition of concentration tails in front of the propagating concentration discontinuity may overcompensate the latter effect and yield a final increase of weight gain rate.

But one also sees that type II diffusion is nothing exceptional requesting any change or modification of diffusion equation. It is a simple consequence of a very rapid change of *P*, i.e., of *S* and *D* with sorbate activity. The increase in *S* is only needed for a rapid increase of penetrant mobility. There is no need for introduction of a new mechanism although a new name may have some practical use. The old Fickian formulation in concentration terms is of course rather inadequate for the description of such less conventional effects because it is applicable only to perfectly ideal material with activity independent sorption. But this limitation was already so thoroughly demonstrated [23] that it has no sense to reopen the subject again.

An important result of this investigation is also the confirmation of the very early finding [13] that the velocity *v* of propagation of concentration discontinuity is not in the slightest manner determined or limited by the diffusion equation and the dependence of *D* on concentration. Experiments, however, very clearly show a drastic increase of *v* with activity of the permeant and temperature of experiment. There is also a substantial dependence of *v* on thermodynamic properties of penetrant and polymer and on mechanical and thermal history of the polymer. Hence one will have to consider the molecular effects connected with swelling much more thoroughly than it was done up till now in order to be able to find the connection between the velocity and the mechanical and thermodynamic properties of the penetrantpolymer system. A next paper will try to discuss some possible approaches to such a molecular theory of unconventional diffusion.

## References

[1] King G (1945). Trans Farad Soc.

[1a] Kishimoto A, Fujita H, Odani H, Kurata M, Tamura M (1960). J Phys Chem.

[2] Alfrey T (1965). Chem Eng News.

[3] Alfrey T, Gurnee EF, Lloyd WGJ (1966). Polymer Sci C.

[4] Michaels AS, Bixler HJ, Hopfenberg HB (1968). J Appl Polymer Sci.

[5] Bray JC, Hopfenberg HB (1969). J Polymer Sci B.

[6] Kwei TK, Zupko HM (1969). J Polymer Sci.

[7] Hopfenberg HB, Holley RH, Stannett V (1969). Polymer Eng & Sci.

[8] Holley RH, Hopfenberg HB, Stannett V (1970). Polymer Eng Sci.

[9] Baird BR, Hopfenberg HB, Stannett V (1971). Polymer Eng Sci.

[10] Kwei TK, Wang TT, Zupko HM (1972). Macromol.

[11] Jaques CHM, Hopfenberg HB, Stannett V (1973). Polymer Eng Sci.

[12] Jaques CHM, Hopfenberg HB, Stannett V (1974). J Appl Polymer Sci.

[13] Peterlin A (1965). J Polymer Sci.

[14] Peterlin A (1969). Makromol Chem.

[15] Frisch HL, Wang TT, Kwei TK (1969). J Polymer Sci A-2.

[16] Wang TT, Kwei TK, Frisch HL (1969). J Polymer Sci A-2.

[17] Hopfenberg HB, Frisch HL (1969). J Polymer Sci.

[18] Hopfenberg HB, Flinn JE (1970). Membrane Science and Technology.

[19] Park GS, Crank J, Park GS (1968). Diffusion in Polymers.

[20] Wang TT, Kwei TK (1973). Macromol.

[21] Kwei TK, Wang TT, Hopfenberg HB (1975). Permeability of Plastic Films and Coatings.

[22] Crank J (1956). Mathematics of Diffusion.

[23] Peterlin A, Hopfenberg HB (1974). Permeability of Plastic Films and Coatings.

